# Mosquito larvae exposed to a sublethal dose of photosensitive insecticides have altered juvenile development but unaffected adult life history traits

**DOI:** 10.1186/s13071-023-06004-8

**Published:** 2023-11-11

**Authors:** Cole J. Meier, Lindsay E. Martin, Julián F. Hillyer

**Affiliations:** Department of Biological Sciences, Vanderbilt University, VU Station B 35-16342, Nashville, TN 37235 USA

**Keywords:** Larvicide, Vector control, Reactive oxygen species, Pest management, Photoactive, Photodynamic, Insecticide resistance

## Abstract

**Background:**

Larvicides are critical for the control of mosquito-borne diseases. However, even sublethal exposure to a larvicide can alter development and life history traits, which can then affect population density and disease transmission dynamics. Photosensitive insecticides (PSIs) are a promising class of larvicide that are toxic when ingested and activated by light. We investigated whether the time of day when exposure occurs, or the process of pupation, affects larval susceptibility to PSI phototoxicity in the mosquito *Anopheles gambiae*, and whether sublethal exposure to PSIs alters life history traits.

**Methods:**

Larvae were treated with lethal concentrations of the PSIs methylene blue (MB) and rose bengal (RB), and larval survival was measured at various times of day. Additionally, larvae were exposed to two concentrations of each PSI that resulted in low and medium mortality, and the life history traits of the surviving larvae were measured.

**Results:**

Pupation, which predominantly occurs in the evening, protected larvae from PSI toxicity, but the toxicity of PSIs against larvae that had yet to pupate was unaffected by time of day. Larval exposure to a sublethal concentration of MB, but not RB, shortened the time to pupation. However, larval exposure to a sublethal concentration of RB, but not MB, increased pupal mortality. Neither PSI had a meaningful effect on the time to eclosion, adult longevity, or adult melanization potential.

**Conclusions:**

PSIs are lethal larvicides. Sublethal PSI exposure alters mosquito development, but does not affect adult life history traits.

**Graphical Abstract:**

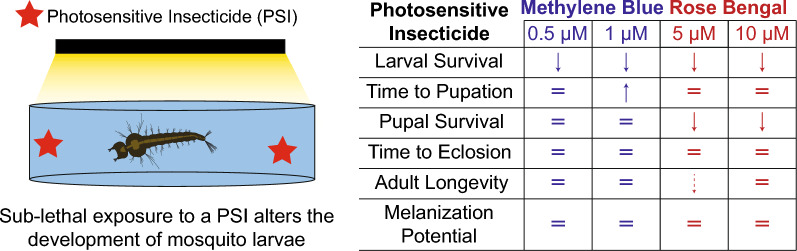

**Supplementary Information:**

The online version contains supplementary material available at 10.1186/s13071-023-06004-8.

## Background

Mosquitoes transmit diseases. Malaria alone—a disease caused by protozoans transmitted by anopheline mosquitoes—killed an average of 700,000 people each year between 2000 and 2021 [1]. A primary method used to reduce the spread of mosquito-borne diseases is the deployment of insecticides that kill adult mosquitoes [2]. Unfortunately, resistance against many adulticides is becoming increasingly prevalent [3–5], and therefore, the concurrent use of larval source management has become critical [6–8].

Larvicides are a highly effective type of larval source management. By targeting the juvenile life stage of the mosquito, larvicides have an intragenerational effect that lowers the density of vector-competent adults, and a transgenerational effect that suppresses the population because killed larvae cannot yield offspring [9]. However, when larvicides are applied to the environment, not all larvae are exposed to a lethal dose, and those that survive can have impacted life history traits [10–13]. For example, *Aedes aegypti* (Culicidae: Culicinae) larvae exposed to a sublethal concentration of malathion developed more slowly and emerge into adults that are more susceptible to Sindbis virus [14]. *Culex quinquefasciatus* larvae exposed to a sublethal concentration of *Cinnamomum verum* oil developed into less fecund adults that laid fewer and less viable eggs [15], and *Anopheles gambiae* (Culicidae: Anophelinae) larvae exposed to a sublethal concentration of the monomolecular surface film Aquatain Mosquito Formulation emerged into adults that were less likely to lay eggs [16]. In contrast to these negative effects, a sublethal exposure of *Ae. aegypti* larvae to Spinosad resulted in larger adults that laid more eggs [17]. This latter beneficial effect shows that exposure to a low dose of an otherwise harmful substance can provide a benefit to the organism, a phenomenon termed hormesis [18–20]. These changes in life history traits following sublethal larvicidal exposure can alter population density and disease transmission dynamics [10, 21–23].

Photosensitive insecticides (PSIs) are a class of larvicides that have received relatively little attention. When PSIs are ingested by a larva and then activated by light, they generate reactive oxygen species (ROS) that indiscriminately damage any macromolecule in their vicinity [9]. When sufficient oxidative damage ensues, the larva dies [24–31]. However, how sublethal PSI exposure affects life history traits remains largely unknown. To our knowledge, only one study [27] has been published on this topic, which found that *Ae. aegypti* exposed to a sublethal concentration of curcumin had delayed development, decreased pupal survival, an altered sex ratio, and reduced adult longevity. Considering that around 180 million years of evolution separate the mosquito subfamilies Culicinae and Anophelinae [32, 33], it is uncertain whether PSIs have similar life history effects across the mosquito lineage. Moreover, given that PSIs are defined by their mode of action and not by their chemical class, it is unknown whether the findings on curcumin for *Ae. aegypti* are representative of what occurs after mosquitoes are exposed to other PSIs.

We investigated whether exposure to the PSIs methylene blue (MB) and rose bengal (RB) alters the development and life history traits of the African malaria mosquito *An. gambiae* (Culicidae: Anophelinae). From the results we can confirm that photoactivated PSIs are lethal to larvae, but reveal that pupation during the photoperiod protects larvae from an otherwise lethal PSI concentration. Moreover, exposure to a sublethal PSI concentration led to altered larval and pupal development, but these effects did not carry across metamorphosis to affect the adult mosquito.

## Methods

### Larval rearing and maintenance

**Fig. 1:**
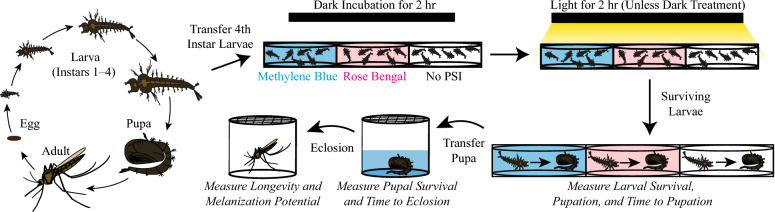
Experimental design for the measurement of *Anopheles gambiae* life history traits following larval exposure to a photosensitive insecticide (PSI)

*Anopheles gambiae* Giles 1902 sensu stricto (G3 strain; Diptera: Culicidae) were raised in an environmental chamber under a controlled 12-h:12-h ambient light:dark cycle at 27 °C and 75% relative humidity. Eggs were hatched in 16″ × 14″ plastic containers with distilled water at a depth of ~ 1.5″, and the larvae were fed a mixture of 2.8 parts koi food to 1 part baker’s yeast. All of the experiments were initiated at the fourth-instar larval stage and were conducted at 27 °C (Fig. 1).

### Incubation of larvae with PSIs, photoactivation, and larval survival

Stock solutions of 2.5 mM trihydrate methylene blue (Sigma-Aldrich, St. Louis, MO) and 2.5 mM disodium salt rose bengal (Thermo Fisher Scientific, Waltham, MA) were prepared by dissolving the chemicals in ultraviolet-sterilized, deionized water. Stock solutions were then wrapped in aluminum foil and stored in the dark, at room temperature.

To expose mosquitoes to PSIs, 10 fourth-instar larvae were added to each well of a clear 6-well plate. As much water as possible was removed, and 5 mL of water containing MB or RB was immediately added. When examining the effects of time of day and pupation on PSI toxicity, lethal concentrations of 20 µM MB and 50 µM RB were used. For the life history experiments, sublethal concentrations of 0.5 µM and 1 µM MB, and 5 µM and 10 µM RB, were used. RB was used at higher concentrations because it is less phototoxic than MB [31]. As an untreated control, larvae were incubated in water without a PSI.

After placing larvae in the PSI solution, the well plate was wrapped in aluminum foil and incubated for 2 h in the dark at 27 °C. For experiments assessing the effects of time of day and pupation on PSI toxicity, larvae were incubated at three different times of day. Zeitgeber notation is used to indicate this, where zeitgeber time (ZT) 0 represents the beginning of a light phase of a 12-h:12-h light–dark cycle. Larvae were incubated beginning at ZT 23 (morning), ZT 5 (afternoon), and ZT 10 (evening). For experiments assessing life history traits, larvae were incubated between ZT 0 and ZT 5.

Following incubation in the dark, the lid of the well plate was removed, and the plate was placed on a white surface that was ~ 15 cm directly underneath a 5000-lumen light-emitting diode (LED) lamp (5000 Lumen Portable LED Work Light; Husky, Pacific, MO). The time when the lamp was turned on was noted as minute 0 of the photoperiod. Larval survival was then monitored every 20 min for 2 h by removing the plate from underneath the lamp, for no more than 2 min, to count surviving larvae. Larval survival was determined via a mechanical stimulus test that used a plastic pipette as a probe; larvae that responded to the stimulus were classified as alive and those that did not were classified as dead. Following the completion of the 2-h photoperiod, the lid was placed back on the well plate and the plate was transferred to the ambient lighting of the environmental chamber. This ambient lighting was insufficiently bright to activate PSIs but sufficiently bright to maintain the mosquitoes’ diurnal cycle.

All of the trials were conducted in duplicate well plates, and for each well plate exposed to a photoperiod, an identical well plate was maintained in darkness (darkness plate) as a control. These darkness plates were sequentially (i) incubated with PSIs for 2 h while wrapped in aluminum foil and survival was measured at the end of the incubation (this coincided with darkness incubation of experimental plates); (ii) covered in aluminum foil for another 2 h and survival was measured again at the end of this second incubation (this coincided with the end of the photoperiod of experimental plates); and (iii) transferred to the ambient light of the environmental chamber [31].

### Larval survival after the photoperiod, and time to pupation

Beginning with the conclusion of the photoperiod (or darkness period when there was no photoperiod), larval survival and time to pupation were measured daily. Larval survival was determined as described above. Pupation was recorded by counting the number of pupae, which were then transferred to ~ 1″-deep water in a container covered with a fine-mesh marquisette top. If there were multiple pupae within a treatment on the same day, they were pooled in the same container. Pupation was measured until all the larvae had either pupated or died. Each day, one drop of larval food was added to each well that contained larvae.

### Pupal survival, time to eclosion, and adult longevity

Pupal survival, time to eclosion, and adult survival were measured daily. Pupal survival was determined by perturbing the water and recording movement; pupae that swam in the water were classified as alive and those that did not were classified as dead. Pupal survival was measured until all of the pupae had either eclosed or died. Time to eclosion was measured as the number of days it took for adult emergence. Adult survival was determined by lightly perturbing the water in the container and recording movement; adults that moved were classified as alive and those that did not were classified as dead. Adult survival was measured until all of the mosquitoes had died. Adults were fed 10% sucrose ad libitum from soaked cotton balls that were changed daily.

### Melanization potential of hemolymph

The melanization potential of the hemolymph in the adults was measured by quantifying the conversion of 3,4-dihydroxy-L-phenylalanine (L-DOPA) to dopachrome by the enzyme phenoloxidase (PO) [34]. In this spectrophotometric assay, the change in optical density at 490 nm (OD_490_) is proportional to the amount of active PO in the hemolymph, and because PO is the rate-limiting enzyme in the melanization pathway, this value represents the melanization potential of the mosquito.

Larvae were incubated in either no PSI, 1 µM MB, or 10 µM RB. For each treatment, half of the larvae were exposed to a photoperiod whereas the other half were not. Larvae were then allowed to pupate and eclose, and hemolymph was collected from adults between 1 and 4 days post-eclosion [34]. For each trial, mosquitoes for each treatment were age-matched. To collect hemolymph, a 0.6-mL microfuge tube with a 5-mm incision at the bottom was nested into a 1.5-mL microfuge tube. Approximately 25 adults were cold-anesthetized, pierced in the thorax with a 0.2-mm-diameter minutien pin, and placed inside the 0.6-mL tube. The nested tubes were then centrifuged at 5000 relative centrifugal force for 5 min at 4 °C, which resulted in the collection of 1–2 µL hemolymph at the bottom of the 1.5-mL tube. Hemolymph samples were immediately stored at -20 °C until further use.

To spectrophotometrically measure melanization potential, each hemolymph sample was diluted 1:50 with sterile deionized water and 10 µL of this solution was added to a cuvette containing 90 µL of 4 mg/mL L-DOPA (Sigma, St. Louis, MO, USA). The OD_490_ was then measured 30 min later, using a BioPhotometer Plus spectrophotometer (Eppendorf, Hamburg, Germany). A negative control that measured the auto-oxidation of L-DOPA was also conducted, where 10 µL of water was added to a cuvette containing 90 µL of 4 mg/mL L-DOPA, and OD_490_ was measured 30 min later. Auto-oxidation of L-DOPA was undetectable.

### Sampling and statistical analysis

For each parameter measured, each treatment was evaluated over a minimum of eight independent trials, using fourth-instar larvae originating from at least two different egg batches. An exception to this was the melanization experiment, where a minimum of four biological replicates were conducted. Each experiment (except melanization) began with at least 120 larvae per treatment, and the exact samples sizes for each parameter measured are presented in the figures. Larvae that died during the dark incubation period were omitted from the analysis. Survival curves were compared using the logrank Mantel Cox test. Time to pupation, time to eclosion, and melanization potential were analyzed using the Mann–Whitney* U*-test. All data analysis was completed in GraphPad Prism version 9.4.1, and statistical differences were deemed significant at *P* < 0.05. Life history trait experiments, except for melanization, were done in sequence. That is, larvae that survived were examined for pupation traits and adult survival. All data collected in this manuscript are included in Additional File 1: Data.

## Results

### Pupation decreases the phototoxicity of PSIs

We previously discovered that it takes around 2 h for larvae to ingest a sufficient quantity of a PSI for it to reach maximal phototoxicity [31]. By using a 2-h incubation and subsequent 2-h photoperiod, we exposed larvae to lethal concentrations of MB and RB and then examined whether PSI toxicity varied across the morning (ZT 23), the afternoon (ZT 5), or the evening (ZT 10).

**Fig. 2A–C:**
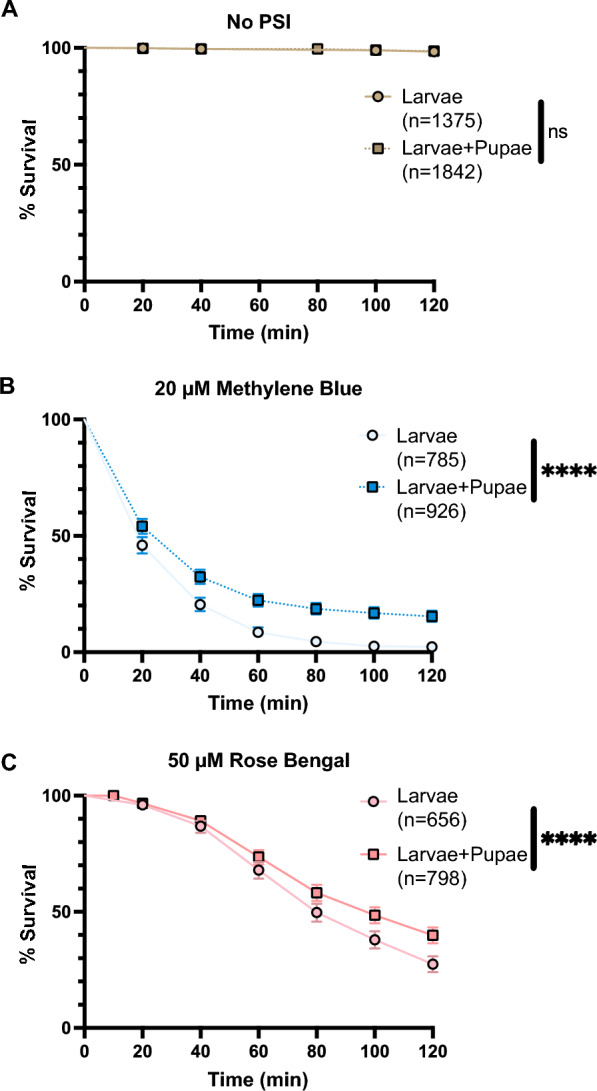
Survival of larvae and pupae following exposure to a PSI and a photoperiod in the evening. In the evening [zeitgeber time (ZT) 10], larvae were incubated either without a PSI (**A**), in 20 µM methylene blue (*MB*) (**B**), or in 50 µM rose bengal (*RB*) (**C**) for 2 h in the dark before survival was measured throughout a 2-h photoperiod. Mosquitoes that pupated during either the darkness incubation or the photoperiod were omitted from the Larvae group, whereas both larvae and those that pupated are included in the* Larvae+Pupae* group. Data were analyzed using the logrank Mantel Cox test.* ns* Not significant *P* > 0.05, ****P < 0.0001. Whiskers indicate the 95% confidence interval (CI).* n* Number of mosquitoes

Time of day did not meaningfully affect the survival of larvae exposed to 20 µM MB or 50 µM RB followed by a photoperiod (Additional File 2: Fig. S1), but pupation during the photoperiod—which almost exclusively occurred in the evening (Additional File 3: Fig. S2)—protected mosquitoes from PSI-mediated death (Table 1). When exposed to 20 µM MB and 50 µM RB and a photoperiod, larval survival was only 6% and 37%, respectively, whereas the survival of larvae not exposed to a PSI was 99%. However, when exposed to MB or RB and a photoperiod, the survival of the mosquitoes that pupated during the experiment was > 96%, which was similar to the survival of pupae not exposed to a PSI. Since ~ 20% of mosquitoes pupated in the evening, we re-analyzed survival by only considering ZT 10 (when pupation was occurring) and compared the survival of larvae to the survival of larvae and pupae combined (larvae plus those mosquitoes that pupated during the experiment). We found that, at the end of the photoperiod, the total survival of mosquitoes (larvae and pupae together) was > 10% higher than the survival of larvae alone (logrank, MB: *χ*^2^ = 72.57, *df* = 1, *P* < 0.0001; RB: *χ*^2^ = 22.55, *df* = 1, *P* < 0.0001; Fig. 2). This indicated that pupation protected the mosquitoes that had been exposed to a PSI.

**Table 1:** Survival of *Anopheles gambiae* larvae and pupae after treatment with methylene blue and rose bengal for 2 h followed by a 2-h photoperiod

	No photosensitive insecticide		Methylene blue (20 µM)		Rose bengal (50 µM)	
	No. mosquitoes	Survival	No. mosquitoes	Survival	No. mosquitoes	Survival
Larvae	9708	99%	2595	6%	2322	37%
Pupae	410	98%	162	96%	161	99%

### Pupation and larval mortality are unaffected by exposure to a photoperiod or an inactive PSI

In preparation for testing whether PSI phototoxicity affects mosquito life history traits, we examined whether a photoperiod or an inactive PSI alters short-term or long-term survival. Based on prior experiments [31], we chose concentrations of each PSI that we predicted would cause low (0.5 µM MB and 5 µM RB) and medium (1 µM MB and 10 µM RB) mortality following photoactivation, and we used these concentrations for the remainder of this study to measure life history traits.

**Fig. 3A–D:**
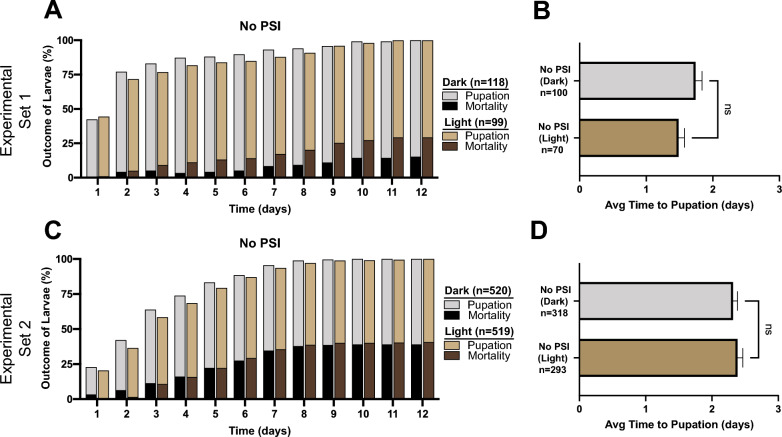
Pupation and mortality of larvae not exposed to a PSI in the presence or absence of a photoperiod. **A, C** Proportion of larvae not exposed to a PSI that pupated or died following exposure to darkness (*Dark*) or a photoperiod (*Light*). **B**, **D** Average (*Avg*) time to pupation for the larvae that pupated in **A** and** C**, respectively. **B**, **D** Data were analyzed using the Mann–Whitney* U*-test (*ns* *P* > 0.05); whiskers indicate the SEM.* n* Number of mosquitoes; for other abbreviations, see Figs.  1 and 2

Exposure to a 2-h photoperiod, but no PSI, did not affect larval survival or time to pupation relative to mosquitoes not exposed to a photoperiod (Fig. 3). Moreover, exposure to a PSI, but no photoperiod, did not affect larval survival or time to pupation relative to mosquitoes not exposed to a PSI (Additional File 4: Fig. S3). The one exception was a 0.5-day increase in the average time to pupation following exposure to 10 µM RB in the dark.

Comparison of the control mosquitoes in the two sets of experiments—low PSI concentration (experimental set 1) and medium PSI concentration (experimental set 2)—showed that the average number of days to pupation for control larvae that were not exposed to either a PSI or a photoperiod was 1.7 days in experimental set 1 but 2.3 days in experimental set 2 (Fig. 3). This difference arose because the experimental sets were conducted several months apart, and used mosquitoes reared at different larval densities, which resulted in some basal differences between the experimental sets. As a result, throughout this study, the data from exposure to low PSI concentration could not be compared to those from exposure to the medium concentration.

### Pupation accelerates following sublethal exposure to MB but not RB

Environmental stressors can have carryover effects that affect development and life history traits [10–20, 23, 35–39]. Considering this, and that pupation protects mosquitoes from MB and RB phototoxicity, we hypothesized that a sublethal PSI exposure accelerates development.

**Fig. 4A–D:**
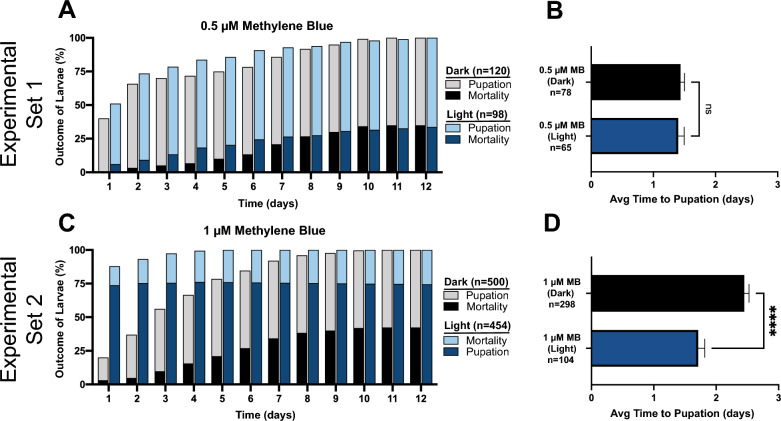
Pupation and mortality of larvae exposed to sublethal concentrations of MB. **A** Proportion of larvae that pupated or died following incubation with 0.5 µM MB and exposure to darkness (*Dark*) or a photoperiod (*Light*). **B** Average days to pupation for the larvae that pupated in **A**. **C** Proportion of larvae that pupated or died following incubation with 1 µM MB and exposure to darkness (*Dark*) or a photoperiod (*Light*). **D** Average days to pupation for the larvae that pupated in **C**. **B**, **D** Data were analyzed using the Mann–Whitney* U*-test (*ns* *P* > 0.05; ****P < 0.0001); whiskers indicate the SEM.* n* Number of mosquitoes; for other abbreviations, see Figs.  1, 2 and 3

We confirmed that photoactivated MB at concentrations of 0.5 µM and 1 µM was toxic to larvae yet left enough larvae alive for the measurement of subsequent development (Fig. 4A, C). Since all of the larvae were incubated for a sufficiently long period of time to internalize MB [31], all of them experienced internal oxidative damage as a result of the photoperiod. However, because more larvae died when exposed to 1 µM MB than when exposed to 0.5 µM MB, we infer that oxidative damage was greater when larvae were exposed to the higher MB concentration. Specifically, the mortality of larvae incubated in 0.5 µM and 1 µM MB was 1% and 12%, respectively, when exposed to a photoperiod, but only 1% and 0% in the absence of a photoperiod. Of the larvae that were exposed to 0.5 µM and 1 µM MB and survived the photoperiod, 10% and 74% died by 1 day later, respectively, and 42% and 74% died by 12 days later (Fig. 4A, C). However, when larvae were not exposed to a photoperiod, incubation in 0.5 µM and 1 µM MB killed 0% and 3% of larvae by 1 day later, respectively, and 35% and 42% by 12 days later (Fig. 4A, C).

Although both concentrations of MB were toxic, only exposure to 1 µM MB followed by a photoperiod led to a change in the time to pupation (Fig. 4B, D). The average time to pupation after exposure to 0.5 µM MB with or without a photoperiod was the same, 1.4 days (Mann–Whitney *U*-test, *U* = 2348, *P* = 0.3733), but the average time to pupation after exposure to 1 µM MB changed from 2.5 days when not exposed to a photoperiod to 1.7 days when exposed to a photoperiod (Mann–Whitney* U*-test, *U* = 10156, *P* < 0.0001). In summary, larvae exposed to 1 µM MB and a photoperiod pupated more quickly, and we speculate that their accelerated development protected these mosquitoes from PSIs.

**Fig. 5A–D:**
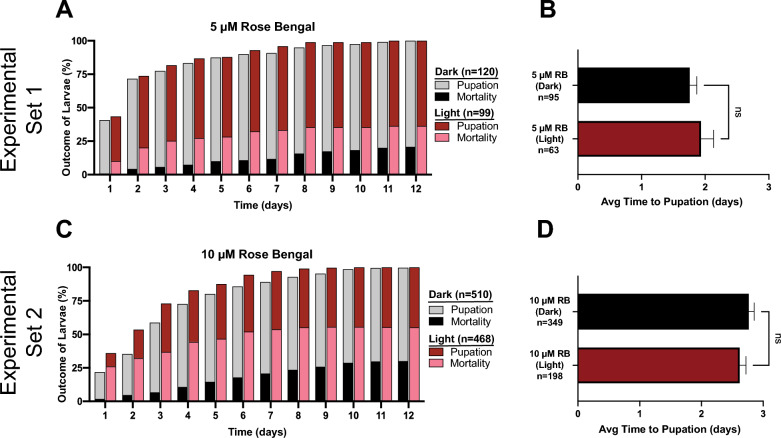
Pupation and mortality of larvae exposed to sublethal concentrations of RB. **A** Proportion of larvae that pupated or died following incubation with 5 µM RB and exposure to darkness (*Dark*) or a photoperiod (*Light*). **B** Average time to pupation for the larvae that pupated in ** A**. **C** Proportion of larvae that pupated or died following incubation with 10 µM RB and exposure to darkness (*Dark*) or a photoperiod (*Light*). **D** Average time to pupation for the larvae that pupated in** C**. **B**, **D** Data were analyzed using the Mann–Whitney* U*-test (*ns*, *P* > 0.05); whiskers indicate the SEM. *n* Number of mosquitoes; for other abbreviations, see Figs.  1, 2 and 3

When we tested RB, both 5 µM and 10 µM RB were phototoxic to larvae while still leaving enough surviving larvae to measure life history traits (Fig. 5A, C). As seen for MB, a higher concentration of RB caused higher phototoxicity. At the conclusion of the photoperiod, the mortality of larvae incubated in 5 µM and 10 µM RB was 0% and 10%, respectively, whereas in the absence of a photoperiod all of the larvae survived. Of the larvae that were exposed to 5 µM and 10 µM RB and survived the photoperiod, 16% and 26% died by 1 day later, respectively, and 46% and 55% died by 12 days later (Fig. 5A, C). However, when larvae were not exposed to a photoperiod, exposure to 5 µM and 10 µM RB killed 0% and 2% of larvae by 1 day later, respectively, and 21% and 30% by 12 days later.

Contrary to what was observed with MB, exposure to RB did not alter the time to pupation (Fig. 5B, D). The average time to pupation after exposure to 5 µM RB was 1.8 days in the dark and 1.9 days in the light (Mann–Whitney* U*-test, *U* = 2996, *P* = 0.9169). At 10 µM RB, the average time to pupation was 2.8 days in the dark and 2.6 days in the light (Mann–Whitney* U*-test, *U* = 32903, *P* = 0.3389).

### Pupal mortality increases following exposure to RB but not MB

**Fig. 6A–F:**
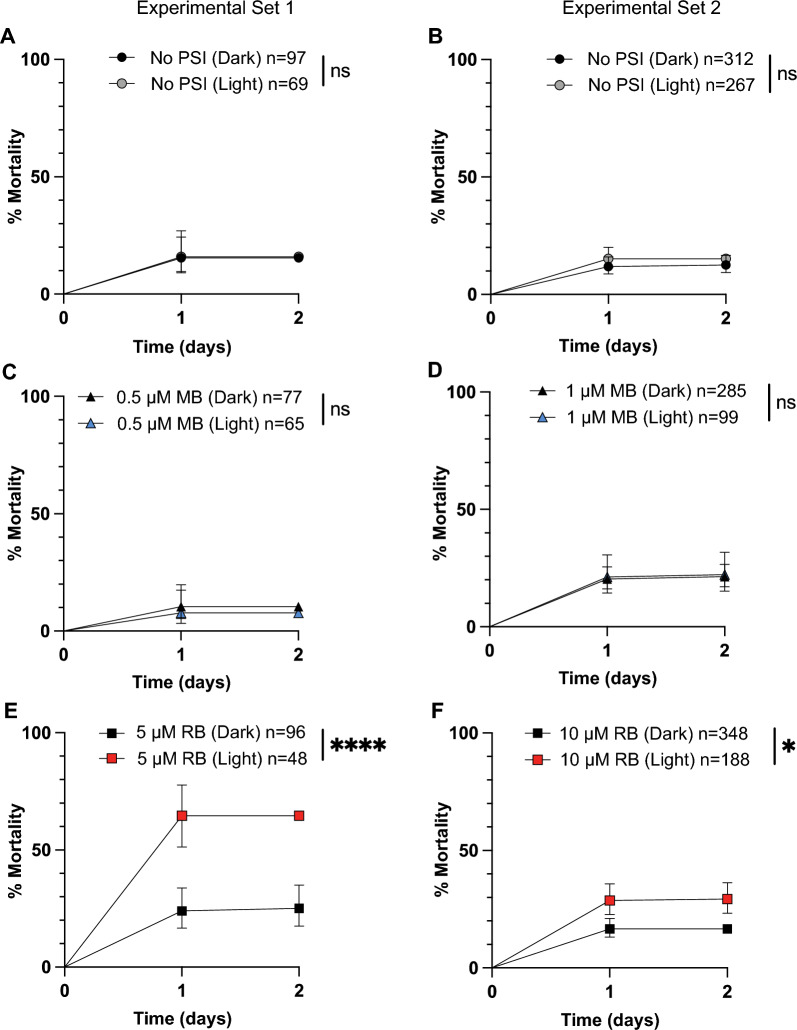
Mortality of pupae that developed from larvae that were exposed to a PSI in the light or dark. Mortality of pupae that were exposed as larvae to either no PSI (**A**, **B**), 0.5 µM MB (**C**), 1 µM MB (**D**), 5 µM RB (**E**), or 10 µM RB (**F**) followed by darkness (*Dark*) or a photoperiod (*Light*). Measurements were made until all of the pupae had either eclosed or died. Data were analyzed using the logrank Mantel Cox test (*ns* *P* > 0.05, * P < 0.05, **** *P* < 0.0001); whiskers indicate the 95% CI. *n* Number of mosquitoes; for other abbreviations, see Figs.  1 and 2

An accelerated pupation timeline may have a cost, and therefore, we hypothesized that pupal mortality would be higher when the larvae had been exposed to 1 µM MB and a photoperiod. To examine this, we tracked pupal mortality in the same populations of mosquitoes that were exposed to MB or RB. The presence or absence of a photoperiod did not affect pupal mortality in larvae that were not exposed to a PSI (Fig. 6A, B), indicating that any effects seen were due to PSI exposure.

Larval exposure to photoactivated MB did not alter pupal mortality (Fig. 6C, D). For pupae that developed from larvae incubated in 0.5 µM MB, mortality was 8% for those exposed to a photoperiod and 10% for those that were not (logrank *χ*^2^ = 0.3062, *df* = 1, *P* = 0.5800). For pupae that developed from larvae incubated in 1 µM MB, mortality was 22% for those exposed to a photoperiod and 21% for those that were not (logrank *χ*^2^ = 0.02908, *df* = 1, *P* = 0.8646). Therefore, neither the internal damage caused by MB photoactivation nor the accelerated time to pupation affected pupal survival.

Contrary to what was observed with MB, larval exposure to photoactivated RB increased pupal mortality (Fig. 6E, F). For pupae that developed from larvae incubated in 5 µM RB, mortality was 65% for those that had been exposed to a photoperiod and 25% for those that had not (logrank *χ*^2^ = 21.30, *df* = 1, *P* < 0.0001). For pupae that developed from larvae incubated in 10 µM RB, mortality was 29% for those that had been exposed to a photoperiod and 17% for those that had not (logrank *χ*^2^ = 11.58, *df* = 1, *P* = 0.0007). Therefore, the internal damage caused by the oxidative stress generated by RB photoactivation led to higher pupal death.

In summary, although larval exposure to photoactivated MB did not affect pupal mortality, larval exposure to photoactivated RB increased pupal mortality. Furthermore, the accelerated pupation rate of larvae treated with 1 µM of photoactivated MB was not accompanied by an increase in pupal mortality.

### Eclosion and adult longevity are unaffected by PSI stress experienced as larvae

**Fig. 7A–F:**
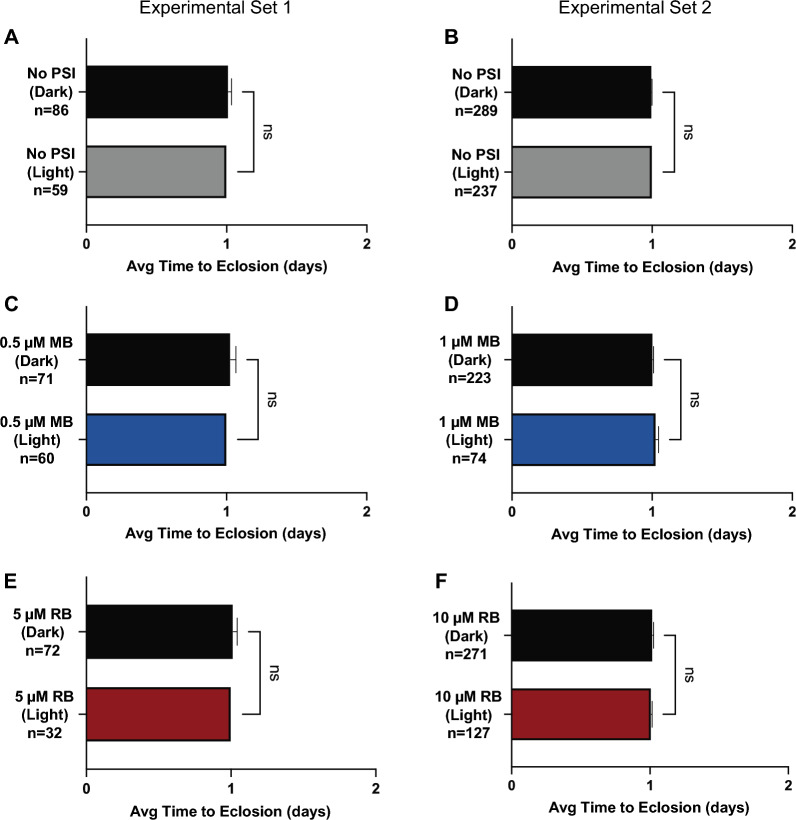
Time to eclosion of pupae that developed from larvae that were exposed to a PSI in the light or dark. Average time to eclosion for pupae that successfully eclosed following larval exposure to either no PSI (**A**, **B**), 0.5 µM MB (**C**), 1 µM MB (**D**), 5 µM RB (**E**), or 10 µM RB (**F**) followed by darkness (*Dark*) or a photoperiod (*Light*). Data were analyzed using the Mann–Whitney* U*-test (*ns* *P* > 0.05); whiskers indicate the SEM. *n* Number of mosquitoes; for other abbreviations, Figs.  1, 2 and 3

**Fig. 8A–F:**
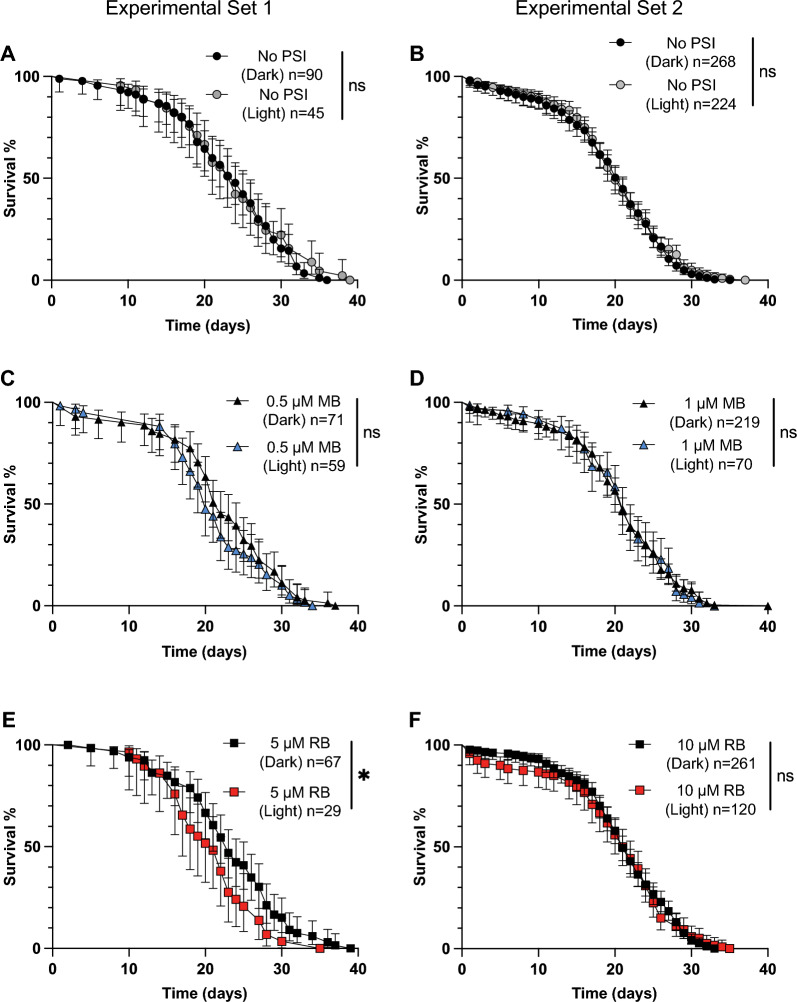
Longevity of adults that eclosed from larvae that were exposed to a PSI in the light or dark. Longevity of adults eclosed from larvae exposed to no PSI (**A**, **B**), 0.5 µM MB (**C**), 1 µM MB (**D**), 5 µM RB (**E**), or 10 µM RB (**F**) followed by darkness (*Dark*) or a photoperiod (*Light*). Data were analyzed using the logrank Mantel Cox test (*ns* *P* > 0.05, * *P* < 0.05); whiskers indicate 95% CI. *n* Number of mosquitoes; for other abbreviations, see Figs.  1 and 2

The effects of larval exposure to environmental stressors can either (i) carry over across metamorphosis to shape adult phenotypes, (ii) decouple across ontogeny to only affect the juvenile life stages, or (iii) affect neither juvenile nor adult phenotypes if conventional recovery occurs [10, 38–40]. Therefore, we used the same population of mosquitoes that developed into pupae to investigate whether larval exposure to a photoactivated PSI affects the time to eclosion or adult longevity. The presence or absence of a photoperiod did not affect the time to eclosion (Fig. 7A, B) or adult longevity (Fig. 8A, B) for mosquitoes that were not exposed to a PSI, indicating that any effects seen were due to PSI exposure.

Larval exposure to MB or RB did not affect the timeline to eclosion (Fig. 7C–F) or adult longevity (Fig. 8C–F) regardless of photoactivation or PSI concentration. For all treatments, the average time to eclosion was 1 day following pupation. The median survival of adults that developed from larvae exposed to 0.5 µM MB and a photoperiod was 20 days; when the photoperiod was omitted, it was 22 days (logrank *χ*^2^ = 1.642, *df* = 1, *P* = 0.2001). The median survival of adults that developed from larvae exposed to 1 µM MB, with or without a photoperiod, was 21 days (logrank *χ*^2^ = 0.04393, *df* = 1, *P* = 0.8340). Similarly, the median survival of adults that developed from larvae exposed to 5 µM RB was 21 days with a photoperiod and 23 days without (logrank *χ*^2^ = 4.415, *df* = 1, *P* = 0*.*0356). Finally, the median survival of adults exposed to 10 µM RB was 21.5 days with a photoperiod and 21 days without (logrank *χ*^2^ = 0.004110, *df* = 1, *P* = 0.9489). Although the two survival curves for 5 µM RB were statistically significantly different, the changes were small. Moreover, because this effect was not repeated at 10 µM, we concluded that the stress experienced by larvae exposed to photoactivated PSI did not carry over and meaningfully affect either the timeline to eclosion or adult longevity.

### Adult melanization potential is unaffected by larval PSI stress

Melanization is a biochemical cascade that mosquitoes deploy as part of their humoral immune response [41, 42]. Melanization is accomplished via the conversion of tyrosine to melanin precursors by PO and other enzymes, followed by the cross-linking of proteins on the surface of a pathogen. This generates ROS such as quinones and semiquinones, which cause oxidative stress that needs to be tolerated by the mosquito [43]. Because photoactivation of a PSI exposes larvae to ROS, we investigated whether a sublethal exposure to photoactivated PSI during the larval stage affects the melanization potential of adult mosquitoes.

**Fig. 9A–C:**
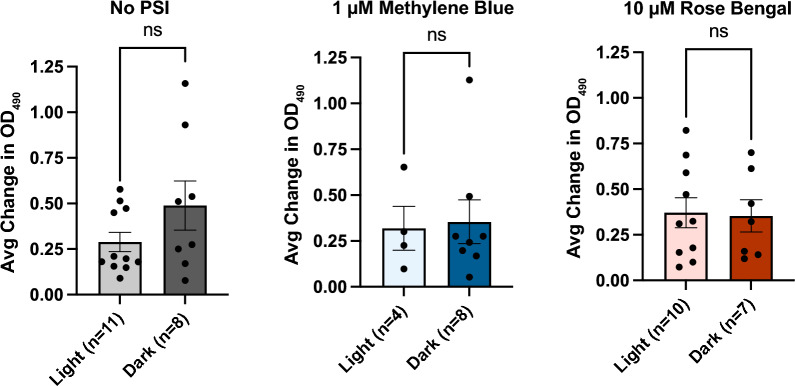
Melanization potential of hemolymph from adults that eclosed from larvae exposed to a PSI. Phenoloxidase activity in the hemolymph of adult mosquitoes that eclosed from larvae exposed to no PSI (**A**), 1 µM MB (**B**), or 10 µM RB (**C**) followed by darkness (*Dark*) or a photoperiod (*Light*). A higher optical density at 490 nm (*OD*_490_) value corresponds to a higher melanization potential. Individual data points are marked with circles. Data were analyzed using the Mann–Whitney* U*-test (*ns* *P* > 0.05); whiskers indicate the SEM.* n* Number of hemolymph samples; for other abbreviations, see Figs.  1, 2 and 3

Measuring the activity of PO—the rate-limiting enzyme in the melanization biochemical cascade—revealed that larval exposure to 1 µM MB or 10 µM RB, with or without a photoperiod, did not affect the melanization potential in the hemolymph of adult mosquitoes (Mann–Whitney *U*-test, NT: *U* = 30, *P* = 0.2723; MB: *U* = 15, *P* = 0.8990; RB: *U* = 35, *P* > 0.9999; Fig. 9). Thus, we concluded that the stress experienced by the larvae exposed to photoactivated MB or RB did not alter the strength of the melanization immune response.

## Discussion

**Fig. 10:**
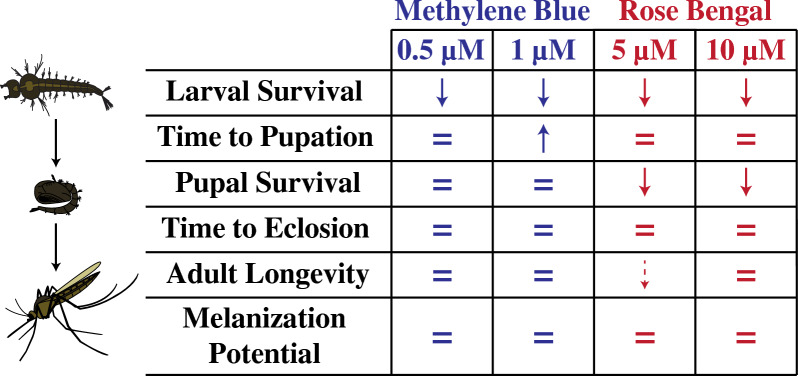
Summary of the effects of exposing larvae to MB and RB followed by a photoperiod

As a holometabolous insect, a mosquito undergoes complete remodeling during its metamorphosis [32, 38]. This allows the mosquito to independently adapt to the different ecological niches that it occupies in its juvenile and adult life stages [40]. However, some environmental factors alter larval development, and these changes can have effects that carry over to modify adult life history traits [10–20, 23, 35–39]. Here, we investigated how exposing mosquito larvae to a sublethal concentration of two PSIs affects development, survival, and melanization potential (Fig. 10). We discovered that exposure to photoactivated MB decreases time to pupation but does not affect pupal survival, whereas exposure to photoactivated RB does not affect time to pupation but decreases pupal survival. Neither PSI meaningfully affects time to eclosion, adult longevity, or adult melanization potential. Altogether, these findings demonstrate that PSIs affect the immature stages of the mosquito, but that these effects do not carry over into adulthood.

PSIs are not just toxic to *An. gambiae*, they are also toxic to *Ae. aegypti*, which is distantly related to *An. gambiae* [31]. *Aedes aegypti* that survived sublethal exposure to the PSI curcumin had delayed development, decreased pupal survival, a lower probability of eclosion, and reduced adult longevity [27]. These detrimental sublethal effects differ from those that we observed after exposing *An. gambiae* to sublethal concentrations of MB and RB. Possible explanations for these different effects include (i) different chemical classes of PSIs have different effects on life history; (ii) members of the two branches of the mosquito lineage to which these species belong—Culicinae and Anophelinae—respond differently to PSIs; or (iii) both of the aforementioned are true. Our earlier finding that the amount of MB or RB required to kill *Ae. aegypti* is less than that required to kill *An. gambiae* suggests that there could be interspecific differences between how a sublethal dose affects life history traits [31]. Importantly, in *An. gambiae*, adult longevity—which is an important determinant of vector competence [21]—is unaffected by larval exposure to a PSI.

Although the effects of both MB and RB are limited to the juvenile mosquito stages, their effects on development differ. Both MB and RB generate ROS in response to light in a process termed intersystem crossing [44–47], yet when these PSIs are ingested by larvae they end up in different locations in the body: MB crosses the midgut epithelium and disperses throughout the hemocoel, whereas RB accumulates in the larval gut [31]. PSI localization within the larvae determines which tissues are damaged upon photoactivation and could explain why MB accelerates pupation but does not affect pupal mortality, whereas RB increases pupal mortality but does not affect the time to pupation. By globally damaging the mosquito, the stress associated with MB exposure may accelerate pupation so that the MB and the damaged tissue in the hemocoel can be shed with the exuviae, thereby normalizing pupal and adult survival. This pupation-mediated shedding scenario is similar to what occurs when carboxylate-modified fluorescent microspheres are injected into the larval hemocoel [48]. However, since RB is confined to the gut, it is unclear whether RB—or the gut damage it causes—is similarly shed during pupation. If it is not, this may hamper pupal development and subsequently lead to an increase in pupal mortality.

An alternative explanation to phenotypic differences following MB and RB exposure is that RB may alter the gut microbial community. PSIs are antibacterial [49–51], and the gut microbiota is critical for insect development [52–56]. Even though pupae do not feed, they do have a pupal microbiome [57, 58], and it is possible that the close contact between RB and the gut microbiota may have a detrimental effect on pupae. Disruption of the gut microbiota by a PSI is not unexpected since other types of pesticides are known to have this effect [51, 59–64]. Therefore, the RB-mediated increase in pupal mortality may be indirectly caused by damage to the mosquito microbiota.

## Conclusions

PSIs, such as MB and RB, are promising larvicides that kill mosquitoes at ecologically relevant concentrations [31]. By generating ROS in response to light, PSIs degrade rapidly upon photoactivation, so they do not accumulate in the environment. Moreover, because of their non-specific mechanism of action, i.e., general oxidative damage, they are less likely to select for resistant mosquito populations [9]. To the best of our knowledge, prior to the present study, it was unknown whether PSIs affect the life history traits of *An. gambiae*. Here, we demonstrated that pupation protects *An. gambiae* larvae from PSI toxicity. Furthermore, larvae that survived sublethal exposure to a PSI had an altered developmental timeline and pupal survival, but the pupae that survived did not carry over detrimental effects into adulthood. Altogether, PSIs are promising larvicides that effectively kill mosquitoes without having hormetic effects on the survivors. Therefore, PSIs are strong candidates for the control of mosquito populations and the curbing of mosquito-borne disease.

### Supplementary Information

**Additional file 1: Data.** Datasets collected for this manuscript.

**Additional file 2: Figure S1.** Larval survival following a photoperiod and photosensitive insecticide (PSI) exposure at different times of the day. In the morning [zeitgeber time (ZT) 23], afternoon (ZT 5) or evening (ZT 10), larvae were incubated in the dark for 2 h in water without a PSI (**A**), in 20 µM methylene blue (MB) (**B**), and in 50 µM rose bengal (RB) (**C**). Larval survival was measured throughout a 2-h photoperiod. Whiskers indicate the 95% confidence interval (CI).* n* Number of mosquitoes.

**Additional file 3: Figure S2.** Pupation of larvae following a photoperiod and PSI exposure at different times of the day. Larvae were incubated in either no PSI, 20 µM MB, or 50 µM RB in the** A**,** B** morning (ZT 23),** C**,** D** afternoon (ZT 5), or** E**,** F** evening (ZT 10). The number of larvae that pupated was measured at the conclusion of the darkness incubation period (0 min), and throughout the succeeding 2-h photoperiod (120 min). Whiskers indicate the 95% CI.* n* Number of mosquitoes.

**Additional file 4: Figure S3.** Pupation and mortality of larvae that were not exposed to a photoperiod.** A** In the absence of a photoperiod (Dark), proportion of larvae that pupated or died following incubation in either no PSI, in 0.5 µM MB, or in 5 µM RB.** B** Average time to pupation for the larvae that pupated in ** A**.** C** In the absence of a photoperiod (Dark), proportion of larvae that pupated or died following incubation in either no PSI, in 1 µM MB, or in 10 µM RB. **D** Average time to pupation for the larvae that pupated in** C**.** B**,** D** Data were analyzed using the Kruskal–Wallis test, followed by Dunn’s multiple comparison test (ns *P* > 0.05, ** *P* < 0.01); whiskers indicate the SEM.* n* Number of mosquitoes.

## Data Availability

The datasets supporting the conclusions of this article are included within the article and its additional files.

## Abbreviations

MB: Methylene blue
PSI: Photosensitive insecticide
RB: Rose bengal
ROS: Reactive oxygen species
ZT: Zeitgeber time

## References

[1] World Health Organization. World malaria report 2022: World Health Organization; 2022. 372 p.

[2] Becker N, Petric D, Zgomba M, Boase C, Dahl C, Madon M (2010). Mosquitoes and their control.

[3] Liu N (2015). Insecticide resistance in mosquitoes: impact, mechanisms, and research directions. Annu Rev Entomol.

[4] Ranson H, Lissenden N (2016). Insecticide resistance in African* Anopheles* mosquitoes: a worsening situation that needs urgent action to maintain malaria control. Trends Parasitol.

[5] Moyes CL, Vontas J, Martins AJ, Ng LC, Koou SY, Dusfour I (2017). Contemporary status of insecticide resistance in the major *Aedes* vectors of arboviruses infecting humans. PLoS Negl Trop Dis.

[6] Tusting LS, Thwing J, Sinclair D, Fillinger U, Gimnig J, Bonner KE (2013). Mosquito larval source management for controlling malaria. Cochrane Database Syst Rev.

[7] World Health Organization. Larval source management: a supplementary measure for malaria vector control. An operation manual: World Health Organization; 2013. 128 p.

[8] Walker K, Lynch M (2007). Contributions of *Anopheles* larval control to malaria suppression in tropical Africa: review of achievements and potential. Med Vet Entomol.

[9] Meier CJ, Rouhier MF, Hillyer JF (2022). Chemical control of mosquitoes and the pesticide treadmill: a case for photosensitive insecticides as larvicides. Insects.

[10] Evans MV, Newberry PM, Murdock CC. Carry-over effects of the larval environment in mosquito-borne disease systems. In: John M, Drake MBB, Michael R Strand, editors. Population biology of vector-borne diseases: Oxford University Press; 2020. p. 155–74.

[11] Muturi EJ, Costanzo K, Kesavaraju B, Alto BW (2011). Can pesticides and larval competition alter susceptibility of *Aedes* mosquitoes (Diptera: Culicidae) to arbovirus infection?. J Med Entomol.

[12] Kesavaraju B, Brey CW, Farajollahi A, Evans HL, Gaugler R (2011). Effect of malathion on larval competition between *Aedes albopictus* and *Aedes atropalpus* (Diptera: Culicidae). J Med Entomol.

[13] Stoks R, Janssens L, Delnat V, Swaegers J, Tüzün N, Verheyen J. Adaptive and maladaptive consequences of larval stressors for metamorphic and postmetamorphic traits and fitness. Development strategies and biodiversity: Darwinian fitness and evolution in the Anthropocene 2022. p. 217–65.

[14] Muturi EJ, Kim CH, Alto BW, Berenbaum MR, Schuler MA (2011). Larval environmental stress alters *Aedes aegypti* competence for Sindbis virus. Trop Med Int Health.

[15] Benelli G, Pavela R, Giordani C, Casettari L, Curzi G, Cappellacci L (2018). Acute and sub-lethal toxicity of eight essential oils of commercial interest against the filariasis mosquito *Culex quinquefasciatus* and the housefly *Musca domestica*. Ind Crops and Prod.

[16] Mbare O, Lindsay SW, Fillinger U (2014). Aquatain® Mosquito Formulation (AMF) for the control of immature *Anopheles gambiae* sensu stricto and *Anopheles arabiensis*: dose-responses, persistence and sub-lethal effects. Parasit Vectors.

[17] Antonio GE, Sanchez D, Williams T, Marina CF (2009). Paradoxical effects of sublethal exposure to the naturally derived insecticide spinosad in the dengue vector mosquito* Aedes aegypti*. Pest Manag Sci.

[18] Guedes RNC, Walse SS, Throne JE (2017). Sublethal exposure, insecticide resistance, and community stress. Curr Opin Insect Sci.

[19] Guedes RN, Cutler GC (2014). Insecticide-induced hormesis and arthropod pest management. Pest Manag Sci.

[20] Cutler CG. Insects, insecticides and hormesis: evidence and considerations for study. Dose Response. 2013;11(2). 10.2203/dose-response.12-008.Cutler PMC3682195 23930099

[21] Shaw WR, Catteruccia F (2019). Vector biology meets disease control: using basic research to fight vector-borne diseases. Nat Microbiol.

[22] Chandrasegaran K, Lahondère C, Escobar LE, Vinauger C (2020). Linking mosquito ecology, traits, behavior, and disease transmission. Trends Parasitol.

[23] Moller-Jacobs LL, Murdock CC, Thomas MB (2014). Capacity of mosquitoes to transmit malaria depends on larval environment. Parasit Vectors.

[24] Dondji B, Duchon S, Diabate A, Herve JP, Corbel V, Hougard JM (2005). Assessment of laboratory and field assays of sunlight-induced killing of mosquito larvae by photosensitizers. J Med Entomol.

[25] de Souza LM, Inada NM, Venturini FP, Carmona-Vargas CC, Pratavieira S, de Oliveira KT (2019). Photolarvicidal effect of curcuminoids from *Curcuma longa* Linn. against *Aedes aegypti* larvae. J Asia-Pac Entomol..

[26] Lima AR, Silva CM, Caires CSA, Prado ED, Rocha LRP, Cabrini I (2018). Evaluation of eosin-methylene blue as a photosensitizer for larval control of *Aedes aegypti* by a photodynamic process. Insects.

[27] Mezzacappo NF, de Souza LM, Inada NM, Dias LD, Garbuio M, Venturini FP (2021). Curcumin/D-mannitol as photolarvicide: induced delay in larval development time, changes in sex ratio and reduced longevity of *Aedes aegypti*. Pest Manag Sci.

[28] Lima AR, Silva CM, da Silva LM, Machulek A, de Souza AP, de Oliveira KT (2022). Environmentally safe photodynamic control of *Aedes aegypti* using sunlight-activated synthetic curcumin: photodegradation, aquatic ecotoxicity, and field trial. Molecules.

[29] Garbuio M, Dias LD, de Souza LM, Correa TQ, Mezzacappo NF, Blanco KC (2022). Formulations of curcumin and D-mannitol as a photolarvicide against *Aedes aegypti* larvae: sublethal photolarvicidal action, toxicity, residual evaluation, and small-scale field trial. Photodiagnosis Photodyn Ther.

[30] de Souza LM, Venturini FP, Inada NM, Iermak I, Garbuio M, Mezzacappo NF (2020). Curcumin in formulations against *Aedes aegypti*: mode of action, photolarvicidal and ovicidal activity. Photodiagnosis Photodyn Ther.

[31] Meier CJ, Hillyer JF (2023). Larvicidal activity of the photosensitive insecticides, methylene blue and rose bengal, in *Aedes aegypti* and *Anopheles* gambiae mosquitoes. Pest Manag Sci.

[32] Misof B, Liu S, Meusemann K, Peters RS, Donath A, Mayer C (2014). Phylogenomics resolves the timing and pattern of insect evolution. Science.

[33] da Silva AF, Machado LC, de Paula MB, da Silva Pessoa Vieira CJ, de Morais Bronzoni RV, de Melo Santos MAV (2020). Culicidae evolutionary history focusing on the Culicinae subfamily based on mitochondrial phylogenomics. Sci Rep.

[34] League GP, Estevez-Lao TY, Yan Y, Garcia-Lopez VA, Hillyer JF (2017). *Anopheles gambiae* larvae mount stronger immune responses against bacterial infection than adults: evidence of adaptive decoupling in mosquitoes. Parasit Vectors..

[35] Murdock CC, Paaijmans KP, Cox-Foster D, Read AF, Thomas MB (2012). Rethinking vector immunology: the role of environmental temperature in shaping resistance. Nat Rev Microbiol.

[36] Vantaux A, Ouattarra I, Lefèvre T, Dabiré KR (2016). Effects of larvicidal and larval nutritional stresses on *Anopheles gambiae* development, survival and competence for *Plasmodium falciparum*. Parasit Vectors.

[37] Brown LD, Shapiro LLM, Thompson GA, Estévez-Lao TY, Hillyer JF (2019). Transstadial immune activation in a mosquito: adults that emerge from infected larvae have stronger antibacterial activity in their hemocoel yet increased susceptibility to malaria infection. Ecol Evol.

[38] Rolff J, Johnston PR, Reynolds S (2019). Complete metamorphosis of insects. Philos Trans R Soc Lond B Biol Sci.

[39] Pechenik JA (2006). Larval experience and latent effects—metamorphosis is not a new beginning. Integr Comp Biol.

[40] Moran NA (1994). Adaptation and constraint in the complex life cycles of animals. Annu Rev Ecol Evol Syst.

[41] Christensen BM, Li J, Chen C-C, Nappi AJ (2005). Melanization immune responses in mosquito vectors. Trends Parasitol.

[42] Hillyer JF (2016). Insect immunology and hematopoiesis. Dev Comp Immunol.

[43] Whitten MMA, Coates CJ (2017). Re-evaluation of insect melanogenesis research: views from the dark side. Pigment Cell Melanoma Res.

[44] Heitz JR. Pesticidal applications of photoactivated molecules. Light-activated pest control. ACS symposium series. 616: American Chemical Society; 1995. p. 1–16.

[45] Amor BT, Jori G (2000). Sunlight-activated insecticides: historical background and mechanisms of phototoxic activity. Insect Biochem Mol Biol.

[46] Barbieri A. Fluorescent sensitising substances as larvicides. The photodynamic action of light. Riv Malariol. 1928;7(4).

[47] Jablonski A (1933). Efficiency of anti-Stokes fluorescence in dyes. Nature.

[48] Brown LD, Thompson GA, Hillyer JF (2018). Transstadial transmission of larval hemocoelic infection negatively affects development and adult female longevity in the mosquito *Anopheles gambiae*. J Invertebr Pathol.

[49] Pham TC, Nguyen VN, Choi Y, Lee S, Yoon J (2021). Recent strategies to develop innovative photosensitizers for enhanced photodynamic therapy. Chem Rev.

[50] Anas A, Sobhanan J, Sulfiya KM, Jasmin C, Sreelakshmi PK, Biju V (2021). Advances in photodynamic antimicrobial chemotherapy. J Photochem Photobiol C: Photochem.

[51] Sperandio FF, Huang Y-Y, Hamblin M (2013). Antimicrobial photodynamic therapy to kill gram-negative bacteria. Recent Pat Antiinfect Drug Discov.

[52] Guégan M, Zouache K, Démichel C, Minard G, Van Tran V, Potier P (2018). The mosquito holobiont: fresh insight into mosquito-microbiota interactions. Microbiome.

[53] Engel P, Moran NA (2013). The gut microbiota of insects—diversity in structure and function. FEMS Microbiol Rev.

[54] Mitraka E, Stathopoulos S, Siden-Kiamos I, Christophides GK, Louis C (2013). Asaia accelerates larval development of *Anopheles gambiae*. Pathog Glob Health.

[55] Coon KL, Vogel KJ, Brown MR, Strand MR (2014). Mosquitoes rely on their gut microbiota for development. Mol Ecol.

[56] Martinson VG, Strand MR (2021). Diet-microbiota interactions alter mosquito development. Front Microbiol.

[57] Moll RM, Romoser WS, Modrzakowski MC, Moncayo AC, Lerdthusnee K (2001). Meconial peritrophic membranes and the fate of midgut bacteria during mosquito (Diptera: Culicidae) metamorphosis. J Med Entomol.

[58] Wang Y, Gilbreath TM, Kukutla P, Yan G, Xu J (2011). Dynamic gut microbiome across life history of the malaria mosquito *Anopheles gambiae* in Kenya. PLoS ONE.

[59] Antonelli P, Duval P, Luis P, Minard G, Valiente MC (2022). Reciprocal interactions between anthropogenic stressors and insect microbiota. Environ Sci Pollut Res.

[60] Widenfalk A, Bertilsson S, Sundh I, Goedkoop W (2008). Effects of pesticides on community composition and activity of sediment microbes–responses at various levels of microbial community organization. Environ Pollut.

[61] Receveur JP, Pechal JL, Benbow EM, Donato G, Rainey T, Wallace JR (2018). Changes in larval mosquito microbiota reveal non-target effects of insecticide treatments in hurricane-created habitats. Microb Ecol.

[62] Songca SP, Adjei Y (2022). Applications of antimicrobial photodynamic therapy against bacterial biofilms. Int J Mol Sci.

[63] Jia Q, Song Q, Li P, Huang W (2019). Rejuvenated photodynamic therapy for bacterial infections. Adv Healthc Mater.

[64] Bara JJ, Montgomery A, Muturi EJ (2014). Sublethal effects of atrazine and glyphosate on life history traits of *Aedes aegypti* and *Aedes albopictus* (Diptera: Culicidae). Parasitol Res.

