# Supplementary material for: Mosquito larvae exposed to a sublethal dose of photosensitive insecticides have altered juvenile development but unaffected adult life history traits
Source: Parasit Vectors. 2023 Nov 11;16:412. doi: 10.1186/s13071-023-06004-8 (PMC10638795; doi:10.1186/s13071-023-06004-8)
Supplement: Supplementary file 2 — Additional file 2: Figure S1. Larval survival following a photoperiod and photosensitive insecticide (PSI) exposure at different times of the day. In the morning [zeitgeber time (ZT) 23], afternoon (ZT 5) or evening (ZT 10), larvae were incubated in the dark for 2 h in water without a PSI (A), in 20 µM methylene blue (MB) (B), and in 50 µM rose bengal (RB) (C). Larval survival was measured throughout a 2-h photoperiod. Whiskers indicate the 95% confidence interval (CI). n Number of mosquitoes. [file 13071_2023_6004_MOESM2_ESM.pdf]

Mosquito larvae exposed to a sublethal dose of photosensitive insecticides have altered juvenile life history traits

Cole J. Meier, Lindsay E. Martin, and Julián F. Hillyer
Department of Biological Sciences, Vanderbilt University, Nashville, TN, USA
julian.hillyer@vanderbilt.edu

Parasites & Vectors, 2023

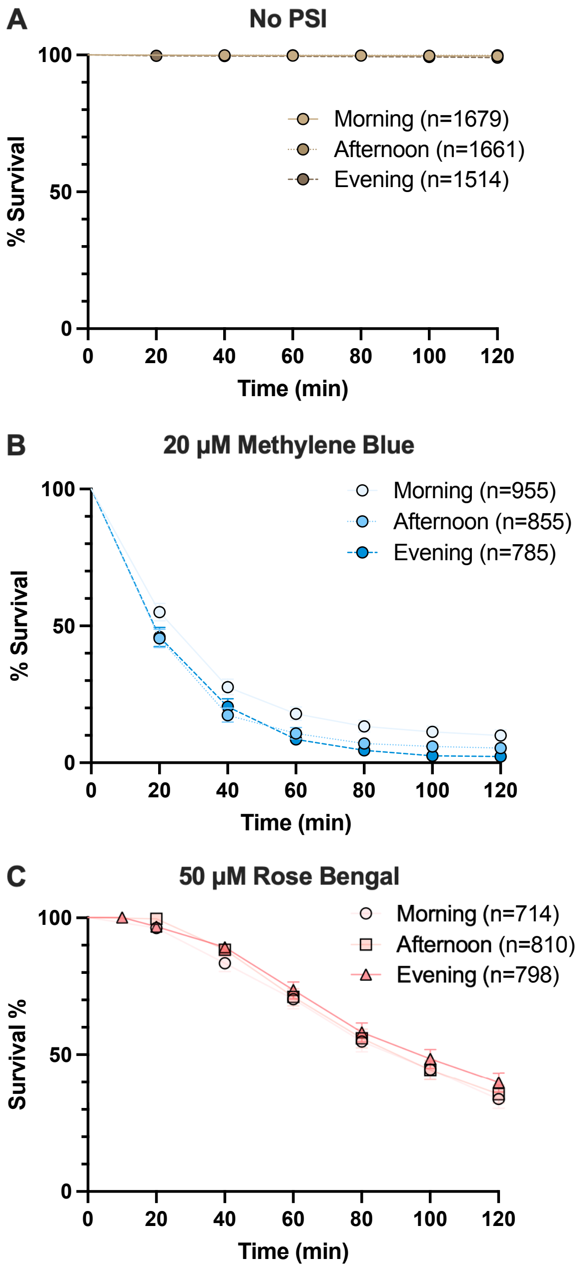

Additional File 2: Fig. S1 Larval survival following a photoperiod and PSI exposure at different times of the day. **(A-C)** In the morning (ZT 23), afternoon (ZT 5) or evening (ZT 10), larvae were incubated in the dark for 2 hr in water without a PSI (A), in 20 µM methylene blue (B), and in 50 µM rose bengal (C). Larval survival was measured throughout a 2 hr photoperiod. Whiskers indicate the 95% confidence interval. ZT, Zeitgeber time n, number of mosquitoes.
