# Supplementary material for: Mosquito larvae exposed to a sublethal dose of photosensitive insecticides have altered juvenile development but unaffected adult life history traits
Source: Parasit Vectors. 2023 Nov 11;16:412. doi: 10.1186/s13071-023-06004-8 (PMC10638795; doi:10.1186/s13071-023-06004-8)
Supplement: Supplementary file 3 — Additional file 3: Figure S2. Pupation of larvae following a photoperiod and PSI exposure at different times of the day. Larvae were incubated in either no PSI, 20 µM MB, or 50 µM RB in the A, B morning (ZT 23), C, D afternoon (ZT 5), or E, F evening (ZT 10). The number of larvae that pupated was measured at the conclusion of the darkness incubation period (0 min), and throughout the succeeding 2-h photoperiod (120 min). Whiskers indicate the 95% CI. n Number of mosquitoes. [file 13071_2023_6004_MOESM3_ESM.pdf]

Mosquito larvae exposed to a sublethal dose of photosensitive insecticides have altered juvenile life history traits

Cole J. Meier, Lindsay E. Martin, and Julián F. Hillyer
Department of Biological Sciences, Vanderbilt University, Nashville, TN, USA
julian.hillyer@vanderbilt.edu

Parasites & Vectors, 2023

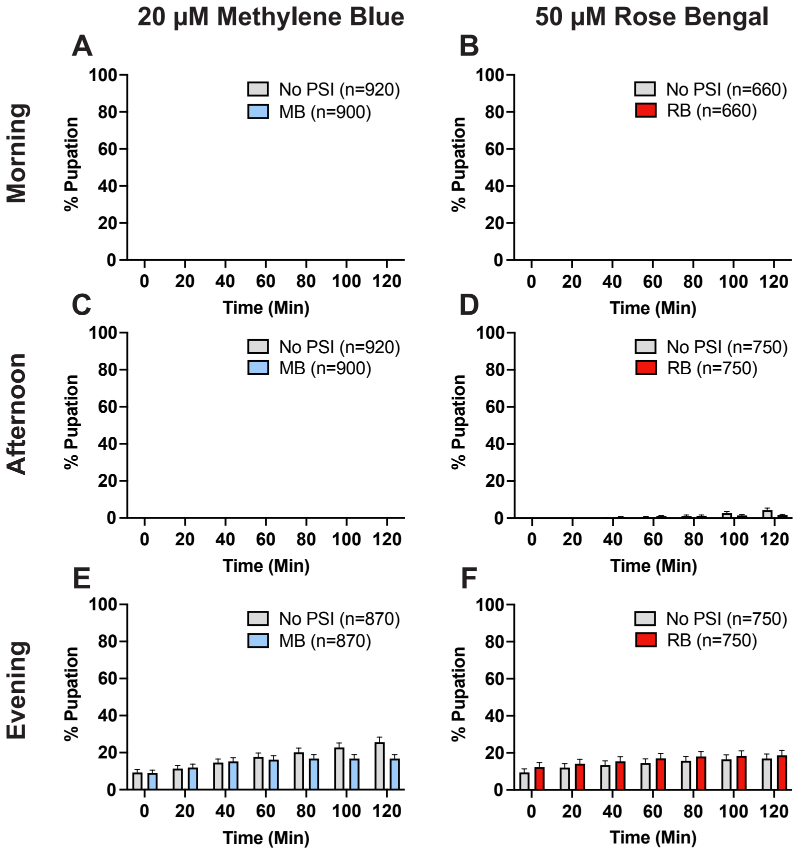

Additional File 3: Fig. S2 Pupation of larvae following a photoperiod and PSI exposure at different times of the day. Larvae were incubated in either no PSI, 20 μM MB, or 50 μM RB in the **(A-B)** morning (ZT 23), **(C-D)** afternoon (ZT 5), or **(E-F)** evening (ZT 10). The number of larvae that pupated was measured at the conclusion of the darkness incubation period (0 min), and throughout the succeeding 2 hr photoperiod (120 min). Whiskers indicate the 95% confidence interval (CI). MB, methylene blue; RB, rose bengal; ZT, Zeitgeber time; n, number of mosquitoes.
