# Supplementary material for: Mosquito larvae exposed to a sublethal dose of photosensitive insecticides have altered juvenile development but unaffected adult life history traits
Source: Parasit Vectors. 2023 Nov 11;16:412. doi: 10.1186/s13071-023-06004-8 (PMC10638795; doi:10.1186/s13071-023-06004-8)
Supplement: Supplementary file 4 — Additional file 4: Figure S3. Pupation and mortality of larvae that were not exposed to a photoperiod. A In the absence of a photoperiod (Dark), proportion of larvae that pupated or died following incubation in either no PSI, in 0.5 µM MB, or in 5 µM RB. B Average time to pupation for the larvae that pupated in A. C In the absence of a photoperiod (Dark), proportion of larvae that pupated or died following incubation in either no PSI, in 1 µM MB, or in 10 µM RB. D Average time to pupation for the larvae that pupated in C. B, D Data were analyzed using the Kruskal–Wallis test, followed by Dunn’s multiple comparison test (ns P > 0.05, ** P < 0.01); whiskers indicate the SEM. n Number of mosquitoes. [file 13071_2023_6004_MOESM4_ESM.pdf]

Mosquito larvae exposed to a sublethal dose of photosensitive insecticides have altered juvenile life history traits

Cole J. Meier, Lindsay E. Martin, and Julián F. Hillyer
Department of Biological Sciences, Vanderbilt University, Nashville, TN, USA
julian.hillyer@vanderbilt.edu

Parasites & Vectors, 2023

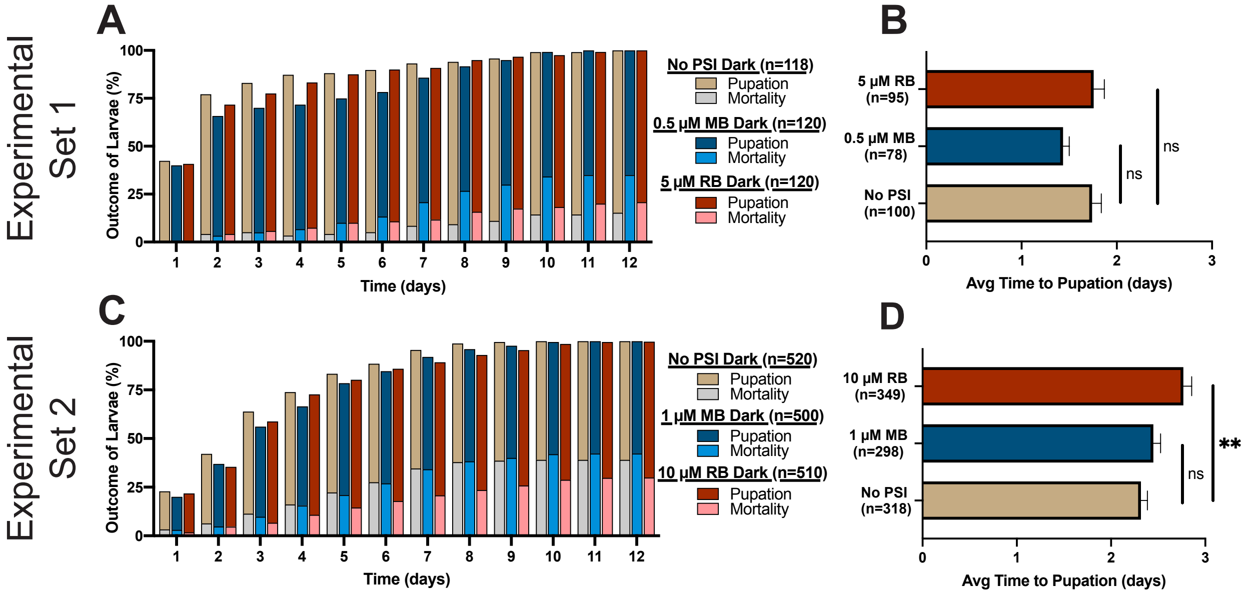

Additional File 4: Fig. S3 Pupation and mortality of larvae that were not exposed to a photoperiod. **(A)** In the absence of a photoperiod (dark), proportion of larvae that pupated or died following incubation in either no PSI, in 0.5 μM MB, or in 5 μM RB. **(B)** Average time to pupation for the larvae that pupated in panel A. **(C)** In the absence of a photoperiod (dark), proportion of larvae that pupated or died following incubation in either no PSI, in 1 μM MB, or in 10 μM RB. **(D)** Average time to pupation for the larvae that pupated in panel C. Data in panels B and D were analyzed using Kruskal-Wallis test, followed by Dunn's multiple comparison test (**, $P < 0.01$; ns, $P > 0.05$), and whiskers indicate the 95% confidence interval (CI). MB, methylene blue; RB, rose bengal; n, number of mosquitoes.
